# On the Role of Auditory Feedback in Robot-Assisted Movement Training after Stroke: Review of the Literature

**DOI:** 10.1155/2013/586138

**Published:** 2013-12-08

**Authors:** Giulio Rosati, Antonio Rodà, Federico Avanzini, Stefano Masiero

**Affiliations:** ^1^Department of Management and Engineering, University of Padova, Via Venezia 1, 35131 Padova, Italy; ^2^Department of Information Engineering, University of Padova, Via Gradenigo 6/A, 35131 Padova, Italy; ^3^Department of Medical and Surgical Sciences, University of Padova, Via Giustiniani 2, 35121 Padova, Italy

## Abstract

The goal of this paper is to address a topic that is rarely investigated in the literature of technology-assisted motor rehabilitation, that is, the integration of auditory feedback in the rehabilitation device. After a brief introduction on rehabilitation robotics, the main concepts of auditory feedback are presented, together with relevant approaches, techniques, and technologies available in this domain. Current uses of auditory feedback in the context of technology-assisted rehabilitation are then reviewed. In particular, a comparative quantitative analysis over a large corpus of the recent literature suggests that the potential of auditory feedback in rehabilitation systems is currently and largely underexploited. Finally, several scenarios are proposed in which the use of auditory feedback may contribute to overcome some of the main limitations of current rehabilitation systems, in terms of user engagement, development of acute-phase and home rehabilitation devices, learning of more complex motor tasks, and improving activities of daily living.

## 1. Introduction

Stroke is the leading cause of movement disability in the USA and Europe [1, 2]. In the EU, there are 200 to 300 stroke cases per 100,000 every year, and about 30% survive with major motor deficits [3]. These impressive numbers are increasing due to aging and lifestyle in developed countries. Improving the outcome of movement therapy after stroke is thus a major societal goal that received a lot of interest in the last decade from many researchers in the medical and engineering fields.

After the acute phase, stroke patients require continuous medical care and rehabilitation treatment, the latter being usually delivered as both individual and group therapy. The rationale for doing motor rehabilitation is that the motor system is plastic following stroke and can be influenced by motor training [4].

Motor learning is a complex process and to date there is still a lack of knowledge on how the sensory motor system reorganizes in response to movement training [5]. Motor learning can be described as “a set of processes associated with practice or experience leading to relatively permanent changes in the capability for producing skilled action” [6]. Early after a stroke, the brain can undergo dramatic plastic changes [7, 8] that can be further enhanced by environmental stimulation. Animal studies have shown that an enriched poststroke recovery environment can induce structural plastic changes in the brain such as decreased infarct volume and increased dendritic branching, spine density, neurotrophic factors, cell proliferation and neurogenesis [9, 10].

The results of rehabilitation on poststroke motor and functional impairment are related to the time between the traumatic event and the beginning of the therapy. Several studies demonstrate that traditional interventions in the acute phase make the recovery of the motor activities easier, since most motor and functional recovery occurs within the first 3 to 6 months poststroke [11, 12].

The final goal of poststroke rehabilitation is to permit patients to independently perform activities of daily living (ADLs), thus facilitating reintegration into social and domestic life, in safe conditions. In this respect, arm and hand function rehabilitation is fundamental and is attracting much attention from the research community.

One currently active research direction concerns the use of novel technological means for the rehabilitation treatment, mainly robotic and virtual reality systems [13]. In most cases, the physical interface is not used in isolation and requires at least a computer interface and possibly a virtual environment to deliver the therapy. The main difference between the two approaches is that robotic systems can actively assist the patient in completing the motor task, while a virtual reality system can only provide the patient with augmented feedback during performance. From this point of view, robot-mediated rehabilitation can be delivered in all phases of the rehabilitation, while virtual reality systems are more likely to be employed in the chronic phase only.

This paper proposes continuous auditory feedback as a novel technology for robot-assisted neurorehabilitation of poststroke patients. As it will be shown, this feedback modality is mostly underexploited in current systems [14], while it can be used to aid user motivation, to improve the motor learning process, and to substitute other feedback modalities in case of their absence.

### 1.1. Robot-Aided Rehabilitation

Three determinants of motor recovery are early intervention, task-oriented training, and repetition intensity [15]. There is strong evidence that highly repetitive movement training, with active engagement by the participant, promotes reorganization and can result in improved recovery after stroke [16, 17]. Robotic devices have the potential to help automate repetitive training after stroke in a controlled fashion and to increase treatment compliance by way of introducing incentives to the patient, such as games [18]. Reinkensmeyer hypothesizes that movement practice with robotic devices can promote motivation, engagement, and effort, as long as some sort of interactive feedback is provided about patient's participation [19]. As an example, the provision of visual feedback that measures participation, such as the size of the contact force against the device, can induce patients to try harder [20]. Moreover, the patient effort can be measured during treatment, together with some kinematic parameters (such as position, velocities, and accelerations), providing a quantitative measure of patient engagement and recovery [21].

Several robotic systems have been recently proposed for use in motor rehabilitation of stroke patients [22]. These can be divided into two main categories: end-effector robots and exoskeletons. A typical example of the former class is the MIT-Manus, the pioneering arm-training planar robot proposed by Krebs et al. [23, 24], where patient-robot contact is at the end-effector level. Exoskeletons are instead wearable robotic devices that can control the interaction at joint level (among others, Pnew-Wrex [25] and Arm-in [26] for the upper limb, and Lokomat [20] and ALEX [27] for the lower limb).

The actual effectiveness of robotic training after stroke is still being discussed. Recent reviews on the first randomized controlled trials (RCTs) showed that patients who receive robot-assisted arm training following stroke are not more likely to improve their activities of daily living (ADLs) with respect to patients who received standard rehabilitation treatment, but arm motor function and strength of the paretic arm may improve [18, 28–30]. Nonetheless, these results must be interpreted very carefully because there are several differences between trials, mainly in the robotic device used, treatment duration, amount of training, type of treatment, and patient characteristics.

One domain to be explored is the role of the robot in acute-phase rehabilitation treatment [31, 32]. According to a recent study on EU centers [33], acute phase patients typically spend >72% of their daily time in nontherapeutic activities, mostly inactive and without any interaction, even though from a plasticity standpoint this time-window is ideal for rehabilitative training [8]. As reported by Mehrholz et al. [28], robotic-assisted training in the acute and subacute phases (i.e., within three months from stroke onset) has a greater impact on the ADLs of participants, compared to therapy in the subsequent chronic phase.

More in general, a key issue is whether robotic systems can help patients learn complex natural movements (like the ones that are typical in the ADLs), instead of training the patient with simple schematic exercises (as with most of the robotic devices developed so far).

One further research challenge concerns the development of home rehabilitation systems, which may help patients continue treatment after hospital discharge [31], as most Healthcare Systems can afford only very short periods of individual therapy in the chronic phase.

### 1.2. Neuroplasticity and Sound

Many recent works in neurosciences suggest that auditory stimulation can enhance brain plasticity by affecting specific mechanisms that contribute crucially to recovery from neurological damage. Brain imaging studies [34] show that neural activity associated with music listening extends beyond the auditory cortex and involves a wide-spread bilateral network of frontal, temporal, parietal, and subcortical areas related to attention, semantic and music-syntactic processing, and memory and motor functions [35, 36], as well as limbic and paralimbic regions related to emotional processing [37–39].

Särkämö et al. [40] suggest that music listening enhances cognitive recovery and mood in poststroke patients. Recent evidence also suggests that listening to enjoyable music unrelated to the cognitive task may even temporarily improve performance in tests of spatial-temporal abilities [41], attention [42], and verbal fluency [43] in healthy subjects.

Interesting results on plasticity have been found in animals subject to exposure to acoustic stimulation. It has been shown that feedback by music stimuli can enhance brain plasticity by (1) increasing neurogenesis in the hippocampus [44], (2) modifying the expression of glutamate receptor GluR2 in the auditory cortex and in the anterior cingulate [45], (3) increasing brain-derived neurotrophic factor (BDNF) levels in the hippocampus [46] and in the hypothalamus [47], and (4) increasing the levels of tyrosine kinase receptor B (TrkB, a BDNF receptor) in the cortex [48]. Changes in glutamate transmission in the peri-infarct area [49] and increased BDNF levels [50] are also crucial plasticity mechanisms that contribute to recovery from stroke. Thus, enhanced cognitive recovery could be attributed to structural plastic changes induced by music stimulation in the recovering brain.

Rapid plastic adaptation due to auditory stimulation is not restricted to cortical motor areas, but it also involves auditory and integrative auditory-sensorimotor circuits [51, 52]. Familiar sounds can facilitate and refine motor responses that have previously been associated with those sounds [53]. Playing music is a particular case of an extremely complex process of integration between the auditory system, proprioceptive feedback and motor control [54]. Positive effects of auditory stimulation in walking abilities of patients with movement disorders have been reported in Parkinson's disease [55] and Multiple Sclerosis [56] or hemiparesis due to stroke [57]. Musical motor feedback can improve the stroke patient's walk in selected parameters (gait velocity, step duration, gait symmetry, stride length and foot rollover path length) compared with conventional gait therapy [58]. Rhythmic sound patterns may increase the excitability of spinal motor neurons via the reticulospinal pathway, thereby reducing the amount of time required for the muscles to respond to a given motor command [59].

Moreover, auditory stimulation increases postural stability in quiet standing tasks and results in a more prominent role for feedback (closed-loop) control over feed-forward (open-loop) control [60].

Plastic adaptation due to auditory stimulation can also induce modifications in the brain overall gross structure [61]. It is known that music practice enhances myelination, grey matter growth and fibre formation of brain structures involved in the specific musical task [62]. Altenmüller [61] asserts that there are two possible explanations why these effects are more pronounced in instrumental music performers than in other skilled activities: first, musical training usually starts very early, sometimes before age six, when the adaptability of the central nervous system is higher; secondly, musical activities are strongly linked to positive emotions, which are known to enhance plastic adaptations. Comparison of the brain anatomy of skilled musicians with that of nonmusicians shows that prolonged instrumental practice leads to an enlargement of the hand area in the motor cortex and to an increase in grey matter density corresponding to more and/or larger neurons in the respective area [63, 64].

### 1.3. Auditory Feedback in Robot-Aided Rehabilitation

This paper analyzes current uses of auditory feedback in poststroke motor rehabilitation and presents strategies for improving robotic and virtual rehabilitation systems by means of leading-edge audio technologies. In this context, the term auditory feedback denotes an audio signal, automatically generated and played back in response to an action or an internal state of the system, understood as both the mechanical device and the user itself. Following this definition, an acoustic or musical stimulus, not automatically generated and not directly related to actions or states, such as sounds aimed at relaxing muscles or music therapy, is not object of this paper.

The design of auditory feedback requires a set of sensors to capture the state of the system, a feedback function to map signals from the sensors into acoustic parameters and triggering events, and a rendering engine to generate audio triggered and controlled by the feedback function. The rendering engine may implement a large set of acoustic signals, from simple sounds or noises to complex music contents.

Feedback may be categorized into either *knowledge of results* (i.e., about the outcome of performing a skill or about achieving a goal) or *knowledge of performance* (i.e., about the movement characteristics that led to the performance outcome) [65, 66]. Informative feedback on errors in movement can (1) facilitate achievement of the movement goal by increasing the level of skill attained or by speeding up the learning process and (2) sustain motivation during learning.

The remainder of the paper is organized as follows.  Section 2 presents relevant approaches, techniques, and technologies from the literature on auditory display and feedback.  Section 3 reviews existing uses of auditory feedback in technology-assisted rehabilitation and shows that its potential is currently not fully exploited in this domain.  Section 4 proposes some innovative uses of auditory feedback in relation to current challenges in robot-assisted movement training.

## 2. Auditory Feedback

Systems for technology-assisted rehabilitation often integrate some form of visual, and possibly multimodal, feedback. While video rendering is a well-studied subject, particularly in the context of virtual rehabilitation, very little attention is devoted to auditory feedback. In this section, we introduce the concept of auditory display and survey relevant approaches, techniques, and technologies available in this domain.

### 2.1. Auditory Display

Auditory display concerns the use of sound to communicate information to the user about the state of a computing device. Mountford and Gaver [67] provide several examples to illustrate the most relevant kinds of information that sound can communicate.

- Information about physical events—we can hear whether a dropped glass has bounced or shattered.
- Information about invisible structures—tapping on a wall helps finding where to hang a heavy picture.
- Information about dynamic change —as we fill a glass we hear when the liquid is reaching the top.
- Information about abnormal structures—a malfunctioning engine sounds different from a healthy one.
- Information about events in space—footsteps warn us of the approach of another person.

Using sound to provide information is appealing for several reasons. First, the bandwidth of communication can be significantly increased, since more than one sensory channel is used. Second, the information conveyed by sounds is complementary to that available visually; thus, sound provides a mean for displaying information that is difficult to be visualised. McGookin and Brewster [68] identified four main ways in which data can be encoded into audio: auditory icons, earcons, speech, and sonification. 

#### 2.1.1. Auditory Icons

**Figure 1:**
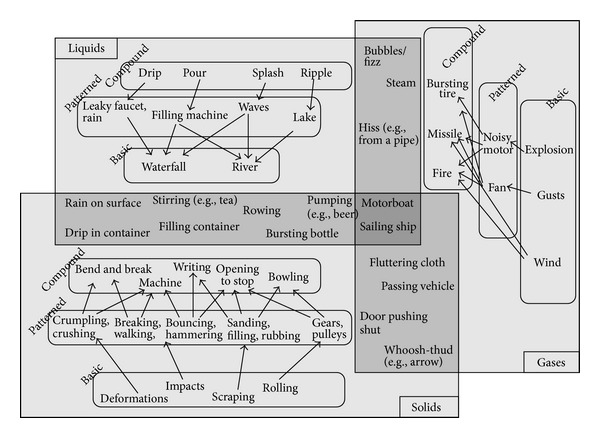
Map of everyday sounds in Gaver taxonomy [69].

Auditory icons are defined by Gaver [69] as “everyday sounds mapped to computer events by analogy with everyday sound-producing events.” One elementary example is the use of a sound of crunching paper to represent an emptying wastebasket. Gaver proposes a variety of algorithms that allow everyday sounds to be synthesized and controlled along meaningful dimensions of their sources. Starting from the three basic physical classes of sound events (solid, liquid, and gas), he identifies several categories of sound events of increasing temporal and spectral complexity, such as breaking, bouncing, spilling, and several more (see Figure 1).

While auditory icons are suited to communicating information where there is an intuitive link between the data and the sound used to represent it, they are less useful in situations where there is no intuitive sound to represent the data.

#### 2.1.2. Earcons

Earcons were originally developed by Blattner and coworkers [70], who defined them as “brief succession of pitches arranged in such a way as to produce a tonal pattern sufficiently distinct to allow it to function as an individual recognisable entity.” Earcons are abstract, synthetic tones that can be used in structured combinations to create auditory messages. Many acoustic and musical features can be used to communicate information by means of structured sounds: pitch, intensity, timbre, rhythm, and space [71].

**Figure 2:**
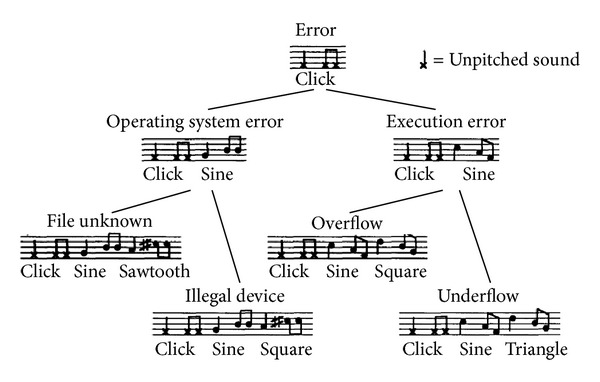
An example of hierarchical earcons proposed by Blattner [70].

As an example, Figure 2 shows a hierarchical menu augmented with earcons: at each level a different auditory cue is used to differentiate alternatives. Earcons must be learned, since there is no intuitive link between the sound and what it represents: they are abstract/musical signals, as opposed to auditory icons.

#### 2.1.3. Sonification

Sonification can be defined as a mapping of multidimensional datasets into an acoustic domain for the purposes of interpreting, understanding, or communicating relations in the domain under study [72]. As such, it can be thought as the auditory equivalent of data visualization.

A particular case of sonification is signal “audification”: this technique amounts to directly translate the signal under consideration into the audio domain, without any additional mapping function. An example of the use of audification is auditory seismology [73], in which seismic waves converted to sound allow to easily recognize relevant aspects of earthquakes including distance, and type of techtonics. Another example is the sonification of information related to the user's physiological state, such as EEG signals or blood pressure [74]. Moreover, auditory feedback has been proved to have interesting potential in the development of on-line BCI interfaces [75].

Sonification is used for nonvisual interface design [76], application interaction control [77], and data presentations [78]. Research has shown that sonification can enhance numeric data comprehension [79] and that listeners can interpret a quick sonified overview of simple data graphs [80]. However, while these techniques are effective in showing trends in large data sets, they are less useful for communicating absolute values.

*Interactive* sonification techniques exploit user movements in the auditory scene. Examples include navigation in sonified graphs and tables [80], and a continuous mouse movement to sonically probe 2D scatterplots [81]. Hunt et al. [82] emphasize that interactive sonification techniques promote user engagement by mimicking interaction with real-world physical objects (e.g., musical instruments). They consequently propose to use high sound complexity, real-time sound generation, and advanced interaction by means of tangible interfaces, rather than standard input devices such as mouse or keyboard.

Several software packages for sonification and auditory display are available [83–85], all of which make different choices about the data formats, the sonification models that are implicitly assumed, and the kinds of allowed interactions.

#### 2.1.4. Speech

Although the focus of this paper is on nonverbal auditory feedback, synthetic speech can also be used for specific purposes, for example, to signal the achievement of a given task. Speech can be advantageous over non-verbal sound in terms of ease of design and learnability. On the other hand, higher-level cognitive processing and more mental resources are required for processing speech: this can be disadvantageous especially in the context of virtual rehabilitation.

Verbal feedback can be used to increase patient's motivation through messages of encouragement and support, similar to those of a human therapist. Moreover, familiar voices (e.g., voices of relatives) may also be presented in order to simulate faithfully a familiar (e.g., domestic) environment. However current text-to-speech systems are intrinsically limited in these respect: synthetic speech is typically obtained through the so-called concatenative synthesis approaches, that is, by concatenating sequences of utterances which have been previously recorded. Natural speech can be obtained only through acquisition of large databases for each voice that has to be synthesized. Current technology does not allow for straightforward “voice cloning”, that is, synthesis of arbitrary verbal messages using an individual's voice.

### 2.2. Sound Spatialization

Spatialization refers to a set of sound processing techniques by which a sound can be virtually positioned in some point of the space around a listener. In the context of auditory display, spatial sound rendering can be used to increase the realism of a virtual environment and to aid user navigation [86]. It can also be employed to aid segregation and recognition of multiple concurrent earcons or sonification streams presented simultaneously in different virtual positions [87].

Spatialization is obtained using either multichannel systems (arrays of loudspeakers) or headphone reproduction. Multichannel systems render virtual sound sources in space using a variety of techniques, including ambisonics and wave-field synthesis [88]. Headphone-based systems have some disadvantages compared to loudspeakers: headphones are invasive and can be uncomfortable to wear for long periods of time; they have nonflat frequency responses; they do not compensate for listener motion unless a tracking system is used. On the other hand, they have two main advantages: they eliminate reverberation and background noise of the listening space and allow to deliver distinct signals to each ear, which greatly simplifies the rendering techniques. Clearly headphone-based systems are advantageous also in terms of costs and flexibility/scalability. 

**Figure 3:**
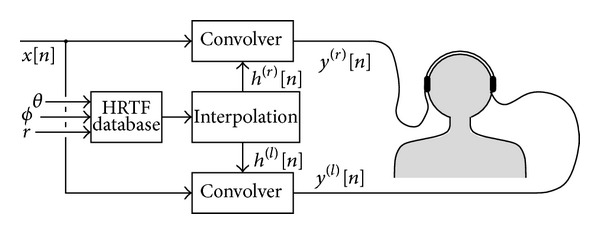
Simplified block scheme of a headphone 3-D audio rendering system based on HRTFs.

Headphone-based 3D audio systems use *head related transfer functions (HRTF)* [89], which depend on the sound source position relative to the listener, and describe how sound is filtered through diffraction and reflection on the head, pinna, and torso before reaching the eardrum. Given the sound source position, binaural signals are synthesized according to the scheme sketched in Figure 3. HRTF sets are typically recorded using “dummy heads”, that is, mannequins constructed from averaged anthropometric measures. Recording individual HRTFs requires special equipment and careful calibration and is an expensive and time-consuming task.

A promising approach is based on simulating the HRTF as a combination of filter blocks that separately account for the effects of torso, head, and pinna [90]. One advantage of these *structural models* is that the filter parameters can in principle be related to individual anthropometric measures (e.g., the interaural distance or the diameter of the cavum conchae) and can thus be adapted to a specific listener [91]. However they have been so far rarely applied in VR applications [92]. 

## 3. Auditory Feedback in Technology-Assisted Rehabilitation and Medical Care

Various typologies of auditory feedback are used in technology-assisted rehabilitation, in the context of both rehabilitation robotics and virtual rehabilitation systems.  Section 3.1 reviews a number of relevant works in these areas.  Section 3.2 focuses exclusively on rehabilitation robotics and presents a comparative analysis of a large number of systems reporting some use of auditory feedback: results of this analysis show that in many cases the potential of auditory feedback is not fully exploited.  Section 3.3 discusses a set of studies in other medical and therapy applications that are nonetheless useful to show possible uses of auditory display in motor rehabilitation. 

### 3.1. Auditory Feedback in Rehabilitation Systems

Audio is used in many rehabilitation systems that utilize game metaphors to motivate patients to perform their tasks. As an example, Cameirão et al. [93] developed a system for the rehabilitation of patients suffering from various neuropathologies such as those brought on by stroke and traumatic brain injury. The system uses a motion capture system with gaming technologies, and audio is employed with a rewarding function: each time the patient intercepts a sphere, this bounces back and the patient receives auditory feedback by means of a “positive sound.” Authors do not specify the nature of the sound, but they suppose that it is a prerecorded, event-triggered sample. Speech and sounds are used also by Loureiro et al. [94] as rewarding feedback to give encouraging words and sounds when the person is trying to perform a task and congratulatory or consolatory words on task completion. 

GenVirtual [95] is another game application devised for patients with learning disabilities. The goal is to aid patients to improve several skills, including motor coordination. The game asks to imitate sounds/color sequences. The user, after hearing/seeing an example, must repeat the sequence by selecting cubes in the virtual environment. Auditory feedback is used to make the sequence memorization easier. With respect to the previous example, sounds are more correlated to user actions (selection of a cube), but again the system uses prerecorded sounds, triggered by a single event. A similar approach, with the addition of sound spatialization, is used by Gil et al. [96]. Their system allows the development of customizable standing exercises. The task is to step over some blocks, which move over a virtual carpet. As the patient steps on the blocks, an accompanying synthetic sound is rendered from the corresponding direction. A game metaphor with auditory feedback is also used by Krebs et al. [97, 98], who developed a virtual environment for stimulating patients to trace trajectories between two or more targets.

Other systems use auditory feedback to reinforce the realism of the virtual reality environment. Johnson et al. [99] simulated a car steering environment for upper limb stroke therapy: the driving scene is completed with auditory, visual, and force feedback in order to render the task a meaningful and functional activity. Boian et al. [100] linked the Rutgers Ankle haptic interface to two virtual environments that simulate an airplane and a boat: weather and visibility conditions can be changed, and sound effects (lightning and thunder) are added for increased realism. Nef et al. [26] experimented the use of the “ARMin” robotic device with several virtual environments, defined by audiovisual cues, which allow the patient to train in activities of daily living like eating, grasping, or playing with a ball. Robot assistance is provided as needed. Hilton et al. [101] use sounds and speech in a virtual environment developed for supporting poststroke rehabilitation and rehearsing everyday activities such as preparing a hot drink. An automatic verbal instruction invites the participant to place the kettle under the tap. If the correct user response is detected, an audiovisual simulation of the activity is played. In all these works, auditory feedback tries to render as realistically as possible the sound of virtual objects present in the scene. However, the interaction with sounds is not realistic, because there is not a continuous relation between user's movements and audio.

In some cases, auditory feedback is used to give information to guide task execution. Masiero et al. [102] developed a robotic device that, during treatment, provides both visual and auditory feedback. Sound intensity is increased to signal the start and the end phase of the exercise, but it is not correlated to patient performance. Nevertheless, the authors report that this feedback was very useful in maintaining a high level of patient attention. In a similar fashion, the 1- and 2-DoF manipulators designed by Colombo et al. [103] provide the patient with visual and auditory feedback both to display the task (start/target positions, assigned path) and to provide feedback about the task execution (start, current handle position, resting phase, and the end of the exercise). If the patient cannot complete the task autonomously, robotic assistance is provided.

One of the few studies that explicitly assess the effectiveness of auditory feedback is due to Schaufelberger et al. [104] (see also [105]), who evaluated the use of earcons to communicate movement related information. In the context of an obstacle scenario, obstacle distance is associated to sound repetition rate, and obstacle height is associated to sound pitch. A study with 17 healthy subjects compared no sound feedback (control condition), distance feedback, height feedback, and combined feedback with a visual feedback presented in all conditions. Results indicate that subjects walk faster and hit fewer obstacles when acoustic feedback is present.

Many other systems include audio, but they do not describe the characteristic of the sound, neither the design criteria. As an example, Wilson et al. [106] report on the development of a virtual tabletop environment for the assessment of upper limb function in traumatic brain injury using an entry level VR system. The system makes use of a Wii interface and auditory feedback, but the paper does not give any other detail on audio cues. Instead, Erni and Dietz [107] give a detailed description of the feedback, but no rationale for the design choices.

### 3.2. Comparative Analysis of Auditory Displays in Rehabilitation Robotics 

The examples discussed above suggest that little attention is devoted to the design and the role of auditory feedback in rehabilitation robotics. In order to quantitatively support this observation, we have analyzed a number of robot-assisted rehabilitation systems that report some use of auditory feedback. Our analysis includes all the papers referenced in recent review articles [108–112], all the papers of a related special issue of the proceedings of the IEEE [31] and the proceedings of two relevant international conferences (ICORR—Int. Conf. on Rehabilitation Robotics, and ICVR—Int. Conf. on Virtual Rehabilitation) since 2006.

**Figure 4:**
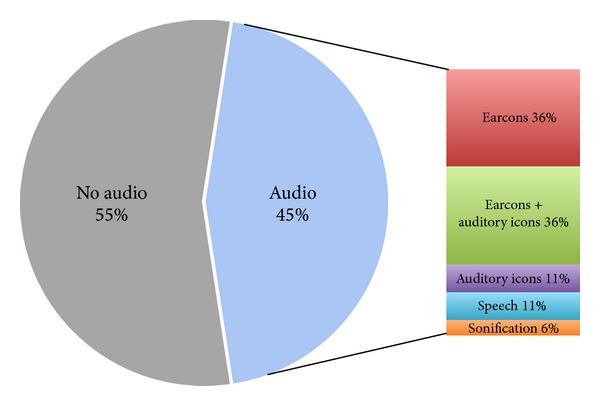
Pie chart representing the distribution of auditory feedback techniques for all the 42 reviewed systems.

A total of 60 papers have been reviewed, related to 42 robot-assisted rehabilitation systems. For each system, we identified the typology of the implemented auditory display, based on the description provided in the papers. The results are summarized in Figure 4. It can be noticed that the majority of the systems do not report any use of auditory feedback. For the remaining ones, the relative majority makes use of earcons, while about one-third implement realistic sounds such as auditory icons or environmental sounds. Little use is made of spatialization and speech, whereas no system implements an auditory display based on sonification. In fact, almost all of the systems make use of very simple sound control (e.g., sound triggered by single events or prerecorded sounds associated to virtual objects).  Table 1 provides additional details about the systems that use some forms of auditory display.

The results of this analysis, together with preliminary experiments such as [113, 114], show that the potential of auditory feedback in rehabilitation systems is largely underestimated in the current literature. In Section 4, we suggest that auditory feedback may be employed in more effective ways, in particular using continuous sonification of user movement.

### 3.3. Auditory Feedback in Other Medical Applications

This section discusses a set of studies that, although not focused on motor rehabilitation, provide nonetheless relevant examples of possible uses of auditory display (Other examples can be found in the large corpus of literature produced by the International Community on Auditory Display (http://www.icad.org/).)

#### 3.3.1. Warnings and Alarms

Auditory displays are widely utilized for warnings and alarms. A newly-released international standard for medical equipment alarms, IEC 60601-1-8, incorporates a long-standing set of investigations about how acoustic alarms should indicate their source (i.e., which equipment has generated the alarm) through distinctive melodies (earcons). Designing earcons in a medical environment presents many problems, such as the need for a prompt identification of simultaneous alarms when multiple concurrent equipment is in function in the same room, and a classification based on the urgency of the alarm. Instead of using melody-based earcons, Sanderson et al. [115] suggest that an alarm with urgency mapped into acoustic features (pitch, speed, or timbre) is more effective in conveying the level of urgency of the event. Edworthy et al. [116] provide a validation study on this design principle.

#### 3.3.2. Continuous Monitoring

In a medical environment, the need to monitor not only alarms, but also different states or levels of physiological signals suggested an extension of earcons, named scalable earcons. Scalable earcons extend the concept of hierarchies of earcons producing intermittent sounds able to represent time-series measurements. A set of scalable earcons to monitor blood pressure was defined by Watson [117], and the results of a test on 24 subjects revealed that scalable earcons can convey a large amount of information with very good accuracy.

Pauletto and Hunt [118] experimented the use of continuous auditory feedback to render acoustically EMG signals from three leg muscles. Real-time auditory display of EMG has two main advantages over graphical representations: it frees the eyes of the physiotherapist, and it can be heard by the patient too who can then try to match with his/her movement the target sound of a healthy person. In [118], each EMG sensor was mapped into the amplitude of a sinusoidal oscillator, and the oscillator frequencies were set in a harmonic relationship with the goal of producing a pleasing sound. The resulting auditory display was validated with a testing group of 21 subjects.

#### 3.3.3. Interfaces for Visually Impaired Users

Auditory display has been used to convey spatial information to subjects with visual impairments. Many studies deal with the use of echolocation devices to provide auditory signals to a user, depending on the direction, distance, and size of nearby objects. Such devices have been studied as prostheses for the blind.

Ifukube et al. [119] designed an obstacle-detection apparatus based on emission of frequency-modulated ultrasounds and detection of reflections from obstacles. Auditory feedback is generated through audification of reflected signals, scaled down to the audible frequency range. Psychophysical experiments showed that auditory feedback is successfully used for the recognition and discrimination of obstacles.

Meijer [120] developed a system for the sonification of a video stream, with applications to vision substitution devices for the blind. An image is sampled from the video stream and converted into a spectrogram in which grey level of the image corresponds to amplitude of spectral components. Although the mapping is highly abstract and unintuitive, users of such device testify that a transfer of modalities indeed takes place.

Talbot and Cowan [121] compared four encodings that allow users to perceive the simultaneous motion of several objects. They compared the results obtained by using various combinations of panning for horizontal motion, pitch for vertical motion (exploiting the so-called Pratt's effect [122]), and sound spatialization through HRTFs. They concluded that users who are blind, or whose visual attention is otherwise occupied, gain information about object motion from an auditory representation of their immediate environment.

## 4. Future Prospects

The analysis in Section 3 shows that in most cases auditory feedback is used as an auditory icon, to signal a special event (e.g., a falling glass) or the existence of an object in the virtual scene. Few systems make use of continuous feedback related to user movements and are mostly based on very basic mapping functions. Only one [123] of the reviewed studies included experiments to verify the actual effectiveness of auditory feedback. Design criteria are never specified.

We are convinced that technology-assisted rehabilitation systems could draw advantage from more acquainted uses of auditory feedback. In this section, we discuss some of such uses, in relation to current research challenges in technology-assisted rehabilitation. Realizing such prospects will require intense experimental work, in order to verify the influence of auditory feedback on motor learning process, as well as its combination with other modalities, such as visual and haptic feedback. Moreover, design criteria have to be defined to choose the suitable auditory cues in relation to a given task.

### 4.1. Presence and Engagement

In Section 1 we emphasized that highly repetitive movement training can result in improved recovery and that repetition, with active engagement by the participant, promotes reorganization. In conventional rehabilitation settings, verbal feedback is extensively used in patient-physiotherapist interaction to increase motivation and to reinforce information to patients. Therefore, supporting engagement and motivation is essential in technology-assisted rehabilitation.

The motivational aspect brought about by auditory feedback during exercise is known and several experiments showed that sound (when related to movements) has benefits which include improved mood, reduced ratings of perceived exertion, and attainment of optimal arousal during physical activity [124]. Moreover, providing a faithful spatial representation of audio events increases the sense of presence, that is, the perception that the virtual environment is real. Hendrix and Barfield [125] report on two studies in which subjects were required to navigate a virtual environment and to complete a questionnaire about the level of presence experienced within the virtual world. Results indicate that the addition of spatialized sound increased significantly one's sense of presence. Rauterberg and Styger [126] carried out an experiment to estimate the effect of auditory feedback on a task-oriented virtual environment. Results indicate that such feedback improves significantly the operator performance and increases positively some mood aspects, such as readiness of endeavour and restfulness.

On the other hand, poorly designed auditory feedback can be counterproductive. If sound is monotonous and little interesting or sound objects are not related to what happens on the virtual scene or again if auditory display is little or not at all informative, users sometimes prefer to eliminate it (for instance, many users disable sound in PC interfaces). Consequently, guidelines to the design of the audio feedback are necessary. This is demonstrated, for example, by Brewster et al. [127] in the case of a sonically enhanced graphical user interface.

Sound quality is another important issue: using psychoacoustic models facilitates the prediction of subjective response to auditory stimuli. Zwicker and Fastl [128] present models of sensory pleasantness, based on a combination of psychoacoustic parameters. Many studies [129] in the field of sound quality indicate that psychoacoustic scales can better predict human evaluation of sound than physical signal measurements.

A third element is the latency of auditory feedback in response to the user's input. Quick and responsive feedback increases user engagement so that users can more easily refine their control activities in the exploration process [130]. Moreover, auditory feedback should be synchronized with other display modalities to allow a coherent unitary percept. Attention must be also paid to the amount of simultaneous sounds presented to the user. While humans are good at selective listening, attending to multiple simultaneous sounds is difficult and the amount of accurate information that can be extracted from simultaneous sound streams is limited [131].

Finally, assessment strategies are necessary. A recent study by Secoli et al. [132] uses distractor tasks to evaluate patient's engagement and effort during a tracking task. Results show that using auditory feedback on tracking error enables patients to simultaneously perform effectively the tracking and a visual distractor task, minimizing the decrease of patient effort that is caused by the distractor when no auditory feedback is given.

### 4.2. Acute Phase Rehabilitation

A major issue in using rehabilitation systems during the acute phase is that acute poststroke patients are lying in bed and are not able to focus attention on a screen. In this case, auditory feedback can be a useful tool, replacing visual displays and integrating haptic feedback.

The possibility of substituting vision with audition is supported by several studies on *sensory substitution*. This concept grounds on the idea that the quality of a sensory modality does not derive from the particular sensory input channel or neural activity involved, but rather from the laws of sensorimotor skills that are exercised, so that it is possible to obtain a visual experience from auditory or tactile input, provided the sensorimotor laws that are being obeyed are the laws of vision. Various studies [119, 120, 133–135] investigate vision substitution with haptics and/or audition through the conversion of video stream into tactile or sound patterns.

Feedback content should be adjusted for the stage of learning of the subject. After stroke, intrinsic feedback systems may be compromised, making it difficult for the person to determine what needs to be done to improve performance. Contrary to vision, we hypothesize that audition produces less dependency on extrinsic feedback and can provide patients with better opportunity to use their own intrinsic feedback [136].

### 4.3. Home Rehabilitation

The use of robotic systems in motor poststroke rehabilitation has many advantages, but the customization and the high costs of these systems make it difficult to carry on therapy after hospital discharge. Home rehabilitation requires low-cost devices and hardware-independent virtual environments. In this context, the audio modality offers interesting opportunities. Audio is usually a low-cost resource with minimum hardware requirements (see, e.g., [137]): medium quality headphone or a common home theater system are enough for almost all applications.

Moreover, audio can integrate more expensive modalities. Properly designed multimodal (haptic-auditory) displays are likely to provide greater immersion in a virtual environment than a high-fidelity visual display alone. This is particularly relevant for low-cost systems, where the quality of the visual display is limited. It is known that the amount of *sensory integration* (i.e., interaction between redundant sensory signals) depends on the features to be evaluated and on the task [138].

Even haptic displays can be compensated by other modalities. Lecuyer [139] showed that properly designed visual feedback can to a certain extent provide a user with haptic illusions, a “pseudohaptic” feedback. Similar ideas are presented in [140] about a cursor interface in which the cursor position is actively manipulated to induce haptic sensations (stickiness, stiffness, mass). The same approach can be experimented with audition and applied to the development of low-cost systems in which pseudohaptic feedback (e.g., inertial effects) is provided via properly designed auditory feedback [141].

Finally, visual and auditory feedback can play a key role in the development of shared user interfaces, creating a trait d'union between acute, postacute, and chronic phase robotic rehabilitation systems and home rehabilitation devices, thus facilitating patient compliance to rehabilitation treatment all along the recovery process.

### 4.4. Motor Learning

From the engineering perspective, in order to optimally stimulate motor learning of the patient after stroke, one should know what kind and amount of stimuli to deliver. As an example, some experimental results suggest that kinematic error drives motor adaptation [142]. This requires control strategies that allow the user to make errors and at the same time to be aware of such errors.

Usually, this kind of information is rendered through visual feedback, that is used to reproduce a virtual task (for instance drag an object and drop it in a box) or to display a marker that moves inside a virtual space and is to be followed by the user. Auditory feedback can be employed to amplify and emphasize small kinematic errors, which are not visible due to limited resolution of video feedback. Also, sound is very suitable to display velocity-related information [143], whose derivation from visual feedback would require a complex elaboration by the patient. Sound can provide information about events that may not be visually attended, and about events that are obscured or difficult to visualize [144]. The integration of audio could also be a promising solution to overcome some common visualization challenges such as visual occlusion, and visually hidden aspects of parallel processes in the background can be made perceptible with auditory feedback.

Comparison with control of upright quiet stance through visual feedback suggests non-redundant roles in multi-sensory integration for the control of posture [145]. Whereas vision provides environmental information and allows prediction of forthcoming events, auditory information processing time is markedly faster than visual reaction times, making it more important for postural reaction to disturbing stimuli.

All these studies suggest that auditory feedback can enhance patient stimulation by providing enriched task related information and performance (error) related cues.

### 4.5. Activities of Daily Living

One of the major goals of poststroke motor rehabilitation is the recovery of the ability to perform ADLs, to facilitate reintegration into social and domestic life. These functional movements typically involve a large number of degrees of freedom of the arm and hand, requiring the development of more sophisticated, multiple DoF robotic therapy devices [146]. Moreover, due to the complexity of the involved motor tasks, their representation to the patient is more challenging than in simple point-to-point rehabilitation exercises, involving just few degrees of freedom of the human arm.

**Figure 5:**
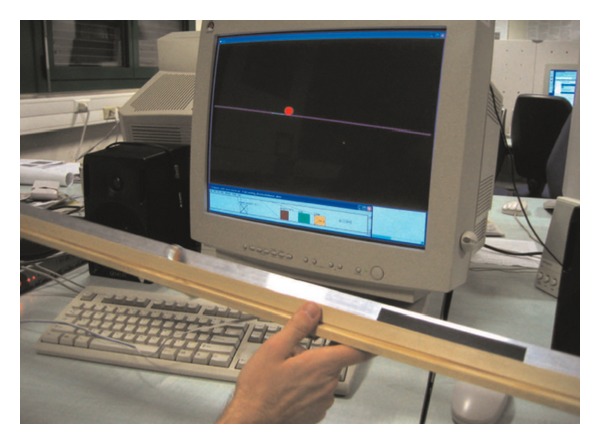
Tilting interface with a glass marble rolling on an aluminum track [143].

During ADLs, our interaction with the world is essentially continuous. Complex tasks, such as walking or riding a bycicle, or even relatively simpler ones, such as reaching and grasping, rely on a mixture of visual, kinesthetic and auditory cues that provide information continuously. As discussed in Section 3, current systems for ADLs training utilize mainly triggered pre-recorded sounds. This approach does not allow to simulate the continuous feedback available in the real world. To this purpose, suitable synthesis models need to be used, which allow a continuous control of audio rendering related to user gestures. An example of continuous, interactive feedback in natural surroundings is represented by the Ballancer [143], a tangible device composed by a 1-meter long track, an accelerometer, and a sonification technique that simulates the motion of a ball on the track. The user has to balance the ball by tilting the track (see Figure 5). The sound of the ball rolling over the track surface is synthesized through a real-time algorithm based on a physical model of the system [147]. User tests show that this continuous auditory feedback decreases the time necessary for balancing the ball. Although not explicitly designed for a rehabilitative scenario, this application demonstrates the potential of continuous sound feedback in supporting complex task learning.

Continuous sonic feedback can also be based on more abstract mappings. In this case, sonification techniques can be used to render auditorily certain cues of complex movements that are hardly reproducible in the visual domain. An interesting related example is presented by Kleiman-Weiner and Berger [148]. They examined the golf swing as a case study, and considered the velocity of the club head and the shoulder rotation relative to the hips as the two most relevant movement cues. These two dimensions were mapped to independent resonant filters to simulate vowel like formants (see the Peterson's vowel chart [149]). In this way the quality of a complex gesture, hardly perceivable through vision due to its high speed, is mapped into a sequence of acoustic vowels, more easily perceivable through audition.

## 5. Conclusion

This paper has reviewed the literature of auditory display with the goal of demonstrating the potential of auditory feedback for robot-aided rehabilitation. The studies discussed in this work show that properly designed auditory feedback can aid user motivation in performing task-oriented motor exercises; can represent temporal and spatial information that can improve the motor learning process; can substitute other feedback modalities in case of their absence. Moreover, the availability of low cost devices to implement auditory feedback makes it particularly suitable for home rehabilitation systems.

In spite of this evidence, very little attention is devoted to auditory feedback in current research on poststroke robot-assisted rehabilitation. Most of the reviewed systems in this paper do not utilize audio, whereas others exploit only a limited set of possibilities, such as earcons or auditory icons. Auditory feedback is mostly implemented in the context of virtual reality systems, to reproduce realistic environmental sounds with the aim of increasing the user's sense of presence. Only in very few cases it is exploited to support the motor learning process, providing an augmented feedback to the user.

A proper use of the auditory sensory channel, supported by further studies on its impact on the motor learning process, is likely to increase the ability of current rehabilitation robotic systems to aid patients learn more complex motion tasks and possibly assist them more effectively in regaining the ability of performing ADLs.

## Figures and Tables

**Table 1:** List of the surveyed robotic/haptic devices that use auditory feedback. Columns 3–7 list the typologies of auditory feedback used, according to the classification of Section 2. Auditory icons include environmental sounds often employed in VR applications.

Reference	Robotic/haptic device	Earcons	Auditory icons	Sonification	Speech	Spatialization
Boian et al. [100], Deutsch et al. [150]	Rutgers Ankle		X			
Colombo et al. [103]	Wrist Rehabilitation Device	X				
Colombo et al. [103]	Shoulder and Elbow Rehabilitation Device	X				
Connor et al. [151]	AFF Joystick	X				
Frisoli et al. [152]	L-Exos	X	X			
Johnson et al. [99]	Driver's SEAT		X			
Wisneski and Johnson [153]	HapticMaster robot (FCS Robotics)	X				
Kousidou et al. [123]	Salford Rehabilitation Exoskeleton			X		
Krebs and Hogan [98]	MIT-MANUS	X				
Loureiro et al. [94]	GENTLE/s				X	
Yeh et al. [154], Stewart et al. [155],	Phantom	X			X	
Nef et al. [26], Staubli et al. [156], Brokaw et al. [157]	ARMin I, II, III	X	X			
Reinkensmeyer et al. [19]	Pneu-WREX	X	X			
Reinkensmeyer et al. [19]	T-WREX	X	X			
Rosati et al. [158]	NeReBot	X				
Shing et al. [159]	Rutgers Master II	X				X
Wellner et al. [160], Koenig et al. [161], Koritnik et al. [105]	Lokomat	X	X			X
